# Polyphasic Characterisation of Non-Starter Lactic Acid Bacteria from Algerian Raw Camel’s Milk and Their Technological Aptitudes

**DOI:** 10.17113/ftb.58.03.20.6598

**Published:** 2020-09

**Authors:** Yasmine Saidi, Beatriz del Rio, Djamel Eddine Senouci, Begoña Redruello, Beatriz Martinez, Victor Ladero, Mebrouk Kihal, Miguel A. Alvarez

**Affiliations:** 1 Applied Microbiology Laboratory, Department of Biology, Faculty of Nature and Life Sciences, University of Oran, 31000 Oran, Algeria; 2 Dairy Research Institute (IPLA-CSIC), Paseo Rio Linares s/n, 33300 Villaviciosa, Spain

**Keywords:** camel’s milk, lactic acid bacteria, molecular identification, acidifying, proteolytic, biogenic amines

## Abstract

**Research background:**

Consumption of spontaneously fermented camel´s milk is common in Algeria, making it a feasible source of diverse lactic acid bacteria (LAB) with the potential to be used as adjunct cultures to improve quality and safety of fermented dairy products.

**Experimental approach:**

Twelve raw camel´s milk samples were used as a source of indigenous LAB, which were further characterised by examining39 phenotypic traits with technological relevance.

**Results and conclusions:**

Thirty-five non-starter LAB (NSLAB) were isolated from 12 Algerian raw camel's milk samples and they were microbiologically, biochemically and genetically characterised. Some isolates showed proteolytic activity, acidifying capacity, the ability to use citrate, and to produce dextran and acetoin. Ethanol, acetaldehyde, methyl acetate, acetoin and acetic acid were the major volatile compounds detected. Cluster analysis performed using the unweighted group with arithmetic average (UPGMA) method, and based on the thirty-nine phenotypic characteristics investigated, reflected the microbial diversity that can be found in raw camel´s milk.

**Novelty and scientific contribution:**

The isolated strains, from a non-typical source, showed interesting technological traits to be considered as potential adjunct cultures. Cluster analysis based on the examined phenotypic characteristics proved to be a useful tool for the typification of isolates when no genetic information is available. These findings may be of use towards an industrialised production of camel's milk dairy products.

## INTRODUCTION

Extensive camel (*Camelus dromedarius*) breeding remains the main agricultural activity of farmers in the arid regions of southern Algeria. Camels are a good source of meat and milk. A female camel may produce from 4 to 14 kg, and sometimes up to 19 kg of milk per day (*1*). It is mainly consumed by the local population in the form of raw milk or as traditional fermented milk. The latter is considered a beverage with interesting health-promoting properties. Fermentation is spontaneous; thus, camel's milk could be a source of LAB (*2*) useful in the more industrialised production of high-quality camel's milk products. Such strains might be better adapted to the camel's milk environment than the LAB commonly used in the dairy industry, which originate from cow's or goat's milk. The isolation and characterisation of indigenous microbial diversity is a key step in order to design tailored starter cultures for artisanal/traditional fermented food that increase the safety and quality of such highly appreciated foodstuffs (*3*).

Non-starter LAB (NSLAB) make an important contribution to the final organoleptic characteristics of fermented milk products; the probiotic properties of some may also be of health benefit (*4*). NSLAB characterisation mainly focuses on their technological properties, such as their proteolytic activity, and their capacity to produce antimicrobial compounds, flavour compounds, and texture components (*5*). However, their production of toxic compounds such as biogenic amines, needs to be eliminated (*6*, *7*). Biogenic amines are low-molecular-mass, nitrogenous, basic organic compounds mainly synthesised *via* the decarboxylation of certain amino acids. Despite the important functions they have in virtually all living organisms, they can accumulate at high concentrations in certain foods, the ingestion of which can be dangerous (*8*). The most important bioactive amines in dairy products are histamine, tyramine, putrescine and cadaverine (*8*), while the main bioactive amine producers are lactobacilli, lactococci and enterococci (*8*).

The present work aims to identify, characterise and typify the cultivable NSLAB isolated from raw camel's milk produced in the western and southwestern Algeria. In order to identify suitable candidates for use as potential adjunct cultures in the manufacture of camel's milk dairy products, isolates were examined for their technological characteristics and their production of bioactive amines.

## MATERIALS AND METHODS

### Milk sample collection

Twelve samples (of about 200 mL each) of camel's milk were collected from eight areas of western and southwestern Algeria (Abadla, Adrar, Bechar, Ghardaia, Mecheria, Oran, Saida and Tindouf), *i.e.* areas with large camel stocks. Milk was obtained directly from the udder of lactating camels. Before collection, the udders were cleaned with sterile warm water. All samples were securely capped, labelled with permanent markers and transported in a cool box at <4 °C to the Applied Microbiology Laboratory, University Oran 1 Ahmed Ben Bella (Oran, Algeria). Classical microbiological analyses were performed upon reception. All chemicals and broths, unless stated otherwise, were purchased from VWR (Barcelona, Spain).

### LAB isolation and growth conditions

Volumes of 10 mL of camel milk were homogenized with 90 mL of 0.1% (*m/V*) sterile peptone water (Oxoid, Basingstoke, Hampshire, UK) to obtain a 1:10 dilution. Tenfold dilutions were then made with the same sterile 0.1% peptone water and 0.1 mL of each dilution plated in duplicate on M17 and MRS agar (Oxoid). After air drying, a second layer of the corresponding medium was poured to generate microaerophilic conditions. For fully anaerobic conditions, the plates were introduced as required into an airtight container with a flame to remove the remaining oxygen. Incubations on M17 were performed at 30 and 45 ºC under microaerophilic conditions, while incubations on MRS were performed at 30 and 45 ºC, at pH=5.4 and 9.6, with 4 and 6.5% NaCl, under microaerophilic and anaerobic conditions. Isolated colonies were streaked twice to ensure they represented pure cultures. Working cultures were kept on MRS or M17 agar slants at 4 °C and streaked every 4 weeks. For long-term storage, stock cultures of the isolates were stored at -20 °C in 30% (*V/V*) glycerol with 70% (*V/V*) skimmed cow's milk (Candia, Oran, Algeria).

### Phenotypic characterisation of the isolates

All isolates were phenotypically assigned to genera on the basis of colony appearance, cell morphology (assessed by microscopy; Zeiss, Göttingen, Germany), Gram staining, catalase activity, spore formation, CO_2_ production from glucose in M17 and MRS broth containing inverted Durham tubes (*9*), the hydrolysis of arginine on M16BCP medium (*10*), growth at 15 and 45 °C, tolerance to 4 and 6.5% NaCl, and tolerance to pH=9.6.

### Molecular identification of the isolates

Total genomic DNA from the isolates was extracted using the GenElute Bacterial Genomic DNA Kit (Sigma-Aldrich, Merck, Madrid, Spain), following the manufacturer’s recommendations. The purified DNA was then stored at 4 ºC until analysis. Purified genomic DNA was used as a template in polymerase chain reaction (PCR) amplification of a fragment of the *16S rRNA* gene in a T100 Thermal Cycler (Bio-Rad, Madrid, Spain). For this, total DNA (1 μL) was used in a final volume of 25 μL containing 2.5 μL buffer 10×, 2.5 μL deoxyribonucleotide triphosphate (dNTPs) 2 mM, 0.2 μL DreamTaq DNA polymerase (Thermo Fisher Scientific, Madrid, Spain), 1 μL of the universal prokaryotic primer S-D-Bact0008-a-S-20 (27F) (5’-AGAGTTTGATCCTGGCTCAG-3’), and 1 μL of the universal prokaryotic primer S-*-Univ1492R-b-A-21 (1492R) (5’-GGTTACCTTGTTACGACTT-3’) (*11*). The PCR conditions were as follows: one cycle at 95°C for 4 min, 35 cycles at 94 °C for 30 s, 50 °C for 30 s and 72 °C for 1.5 min, and a final extension step at 72 °C for 7 min (*12*). Amplicons were purified using the ATP™ Gel PCR Fragment DNA Kit (ATP Biotech Inc., Taipei, Taiwan) and sequenced using the 27F primer from Macrogen (Amsterdam, The Netherlands). The obtained sequences were compared with those in the GenBank database using BLAST suite software (*13*). Partial sequencing of the superoxide dismutase gene (*sod*) from *Enterococcus* isolates to identify them as either *Enterococcus durans, Enterococcus hirae* or *Enterococcus faecium*. Total DNA was used as a template for PCR amplification of an internal fragment of *sod*. The PCR reaction was performed as described above but using the degenerated primers *sodAd1* (5′-CCITAYICITAYGAYGCIYTIGARCC-3′) and *sodAd2* (5′-ARRTARTAIGCRTGYTCCCAIACRTC-3′) (*14*). The PCR reaction conditions included a denaturation step (3 min at 95 °C), followed by 35 cycles of amplification (30 s of denaturation at 95 °C, 30 s of annealing at 42 °C, 90 s of elongation at 72 °C), and a final extension step (7 min at 72 °C). Amplicons were purified using the ATP™ Gel PCR Fragment DNA Kit and sequenced using the primer *sodAd1* from Macrogen. The obtained sequences were compared with those in the GenBank database using BLAST software (*13*).

### Technological characterisation

Technological characterisation of the isolated lactic acid bacteria has been assessed by the determination of acidifying capacity, and the proteolytic activity, the citrate utilisation, the acetoin, dextran, volatile compounds and antimicrobial substances production. The following assays were performed in triplicate unless otherwise stated.

#### Determination of acidifying capacity

Overnight cultures of the isolates were used to inoculate ultrahigh temperature (UHT) skimmed cow's milk at 1% (*V/V)*, incubated at 30 °C). The change in the pH was recorded every 30 min for 18 h with a pH meter (Orion™ Versa Star™, Thermo Fischer Scientific, Madrid, Spain) only values after 6 and 18 h are shown. Milk clotting was assessed at the end of fermentation.

#### Determination of proteolytic activity

The proteolytic activity of the isolates was examined by a qualitative method on plate count agar (PCA; Oxoid) supplemented with 2% UHT skimmed cow's milk (Oxoid). Isolated cultures were streaked on these plates and incubated at 30°C for 24h. A clear zone around the colonies indicated proteolytic activity. In addition, the proteolytic activity of the isolates was quantitatively determined using the *o*-phthaldialdehyde (OPA) method (*12*). Briefly, after incubation of the strains in the same type of skimmed cow's milk at 30 °C for 24h, the protein fraction was precipitated out of 1-mL samples by adding 2 mL of 0.75 M trichloroacetic acid (Sigma-Aldrich, Merck) and 0.2 mL of water. The mixtures were then vortexed for 2 min and filtered through a 0.20-μm pore diameter membrane (Millipore, Bedford, MA, USA). The OPA reagent (Sigma-Aldrich, Merck) was added to the filtrates and the absorbance was measured at 340 nm using a Benchmark Plus Microplate Spectrophotometer (Bio-Rad, Hercules, CA, USA). Results were expressed as glycine (Gly) using an appropriate calibration curve (concentration range 0.1–10 mM). Positive controls were established by inoculating UHT skimmed cow's milk with *Lactococcus lactis* NCDO 604^T^ and *Lc. lactis* SH4109 (*15*) strains known for their strong proteolytic activity. Non-inoculated UHT skimmed milk samples were incubated under the same conditions as negative controls.

#### Citrate utilisation

Kempler and McKay (KMK) culture medium containing iron(III) citrate and potassium hexacyanoferrate(III) (Biochem Chemopharma, Cosne-Cours-sur-Loire, France) was used to assess the capacity of the isolates to utilise citrate (*16*). Cultures that turned blue after incubation at 30 or 45 °C for 24-48 h were considered able to use citrate.

#### Production of acetoin

Acetoin production was tested by inoculating the isolates into 10 mL of Clark and Lubs medium, incubating at 30 and 45 °C for 24 h, and testing *via* the Vosges-Proskaur (VP) reaction by adding to1 mL of the culture 0.5 mL of the VP I reagent (prepared by adding α-naphtol to absolute alcohol to a volume fraction of 6%) and 0.5 mL of the VP II reagent (a 16% solution of NaOH prepared in distilled water). The reaction tubes were then agitated for 1 min. After a 10-minute rest, the presence of a pink ring on the surface of the culture was deemed to indicate the production of acetoin.

#### Production of dextran

Dextran production was investigated on Mayeux, Sandine and Elliker (MSE) agar medium rich in sucrose. After an incubation at 30 or 45 °C for 24-48 h, viscous colonies were deemed to be those of dextran producers.

#### Production of volatile compounds

The production of volatile compounds was assessed by inoculating UHT skimmed cow's milk with overnight cultures at 1% (*V/V*). After 24 h of incubation at 30 °C, the volatile compounds were quantified using a head space gas chromatograph (Agilent Technologies, Wilmington DE, USA) connected to a mass spectrophotometer detector (HS/GC/MS) equipped with a DB-WAXetr capillary column (60 mm×0.25 mm×0.25 μm, Agilent). Sample preparation and gas chromatographic separation were performed as previously described (*17*). Compounds were quantified as the normalized value of their chromatogram peak area using cyclohexanone (3.6 μg/mL) as an internal standard, which was given a value of 100 (*12*). Non-inoculated UHT skimmed cow's milk samples were incubated under the same conditions as negative controls. The difference between the values obtained for the sample and negative controls were calculated.

#### Determination of the production of antimicrobial substances

The antibacterial activity of the isolates was determined *via* well diffusion assays as reported by Schillinger and Lücke (*18*). *Lactobacillus sakei* CECT 906^T^, *Lactococcus lactis* ssp. *cremoris* MG1363, *Listeria innocua* CECT 910^T^, *Micrococcus luteus* NCIMB 8166, *Streptococcus thermophilus* LMD9 and *S. thermophilus* CNRZ 1066 were used as microbial indicators. Briefly, 45 mL of 1.2% agar medium at 45 °C were vigorously mixed with 40μL of an overnight culture of each indicator and poured into Petri dishes. Supernatants from overnight cultures, in duplicate, of the tested strains were adjusted to pH=6.57.0 with 0.1 M NaOH, and filtered through a 0.20-μm pore diameter membrane (Millipore). Aliquots of 40 μL of each supernatant were placed in a 4-mm well excavated in the agar plates. Antimicrobial activity was determined by measuring the diameter of the inhibition halo after 24 h of incubation under appropriate conditions. A halo diameter of >8 mm was considered a positive result. The average result of duplicate assays is shown.

#### Production of biogenic amines

Biogenic amine production was measured in supernatants obtained after 48 h of incubation of the strains in 10 mL of MRS broth supplemented with either 1 mM tyrosine, 1 mM histidine, 1 mM ornithine, 1 mM agmatine (ornithine and agmatine are precursors of putrescine *via* different pathways) or 1mM lysine (all from Sigma-Aldrich, Merck). Tyramine, histamine, putrescine and cadaverine were detected as previously described (*7*). Briefly, 100 μL of supernatant, obtained after centrifugation of the cultures at 3000×*g* for 10 min (5424 benchtop centrifuge; Eppendorf, Hamburg, Germany), were derivatised with diethyl ethoxymethylenemalonate (Sigma-Aldrich, Merck) following a previously described protocol (*19*). Derivatised samples were filtered through a 0.2-μm pore diameter membrane and analysed by ultrahigh performance liquid chromatography (UHPLC) using a Waters H-Class ACQUITY UPLC apparatus with a UV detector (Waters, Milford, MA, USA) controlled by Empower v. 2.0 software (*20*) (Waters) under the conditions described by Redruello *et al.* (*19*).

### Cluster analysis

The relationships among isolates were examined by cluster analysis using the unweighted group with arithmetic average (UPGMA) method, and based on their physiological (production of CO_2_, hydrolysis of arginine, growth at 45°C, pH=5.4 and 9.6, tolerance of 4 and 6.5% NaCl), technological (reduction of pH, clotting of milk, proteolytic activity, ability to utilise citrate, production of dextran and acetoin, production of volatile compounds and antimicrobial activity) and safety (biogenic amine production) traits. A dendrogram was constructed to reflect inter- and intraspecies differences. Analyses were performed using the Dendro UPGMA server (*21*). The final tree was generated on the iTOL webpage (*22*).

## RESULTS AND DISCUSSION

### Phenotypic identification of strains

All 35 isolates were Gram-positive, catalase-negative and non-spore forming, and thus, considered to be LAB (Table 1). The morphological distribution (microscopic observation) indicated 21 isolates (60%) to be ovococci, 7 (20%) coccobacilli, and another 7 (20%) rods.

**Table 1:** Phenotypic and genotypic identification of lactic acid bacteria isolated from Algerian camel's milk

Isolate	Species (based on molecular identification)	CO_2_ production	Arginine hydrolysis	Growth at (NaCl)/%				
*t*=45 °C	pH=5.4	pH=9.6	4	6.5
LEY1	*Leuconostoc mesenteroides*	+	-	-	-	-	+	-
LEY2	*Leuconostoc mesenteroides*	+	-	-	-	-	+	-
LEY3	*Leuconostoc mesenteroides*	+	-	-	-	-	+	-
LEY4	*Leuconostoc mesenteroides*	+	-	-	-	-	+	-
LEY5	*Leuconostoc mesenteroides*	+	-	-	-	-	+	-
LEY9	*Leuconostoc mesenteroides*	+	-	-	-	-	+	-
LEY10	*Leuconostoc mesenteroides*	+	-	-	-	-	+	-
LEY14	*Lactobacillus rhamnosus*	-	+	+	+	-	+	-
LEY15	*Lactobacillus rhamnosus*	-	+	+	+	-	+	-
LEY16	*Lactobacillus rhamnosus*	-	+	+	+	-	+	-
LEY17	*Lactobacillus rhamnosus*	-	+	+	+	-	+	-
LEY18	*Lactobacillus rhamnosus*	-	+	+	+	-	+	-
LEY19	*Lactobacillus rhamnosus*	-	+	+	+	-	+	-
LEY20	*Lactobacillus rhamnosus*	-	+	+	+	-	+	-
LMA16	*Enterococcus hirae*	-	+	+	-	+	+	+
LMA18	*Enterococcus hirae*	-	+	+	-	+	+	+
LMA1	*Enterococcus faecium*	-	+	+	-	+	+	+
LMA2	*Enterococcus faecium*	-	+	+	-	+	+	+
LMA3	*Enterococcus faecium*	-	+	+	-	+	+	+
LMA4	*Enterococcus faecium*	-	+	+	-	+	+	+
LMA5	*Enterococcus faecium*	-	+	+	-	+	+	+
LMA6	*Enterococcus faecium*	-	+	+	-	+	+	+
LMA7	*Enterococcus faecium*	-	+	+	-	+	+	+
LMA8	*Enterococcus faecium*	-	+	+	-	+	+	+
LMA9	*Enterococcus faecium*	-	+	+	-	+	+	+
LMA10	*Enterococcus faecium*	-	+	+	-	+	+	+
LMA11	*Enterococcus faecium*	-	+	+	-	+	+	+
LMA12	*Enterococcus faecium*	-	+	+	-	+	+	+
LMA13	*Enterococcus faecium*	-	+	+	-	+	+	+
LMA14	*Enterococcus faecium*	-	+	+	-	+	+	+
LMA15	*Enterococcus faecium*	-	+	+	-	+	+	+
LMA17	*Enterococcus faecium*	-	+	+	-	+	+	+
LMA19	*Enterococcus faecium*	-	+	+	-	+	+	+
LMA20	*Enterococcus faecium*	-	+	+	-	+	+	+
LMA21	*Enterococcus faecium*	-	+	+	-	+	+	+

The isolates of ovococci were homofermentative and able to grow at 45 ºC in the presence of 6.5% of NaCl, and even at pH=9.6 (Table 1), suggesting they belonged to *Enterococcus* genus. Since none of the coccobacilli were able to hydrolyze arginine, they were assigned to the genus *Leuconostoc* (Table 1) (*23*, *24*). All 7 rod-shaped isolates were presumptively classified within *Lactobacillus* genus.

### Results of molecular identification of isolates

Once phenotypically characterised, all 35 isolates were identified at the species level by sequencing and inspection of the *16S rRNA* gene. For isolates belonging to *Enterococcus*, the *sod* gene was also sequenced and compared. The results of the molecular identification agreed with those of the phenotypic identification at the genus level. Table 1 shows that the 35 isolates belonged to four species. The most common was *Enterococcus faecium* (54%), followed by *Leuconostoc mesenteroides* and *Lactobacillus rhamnosus* (20% each), and then *Enterococcus hirae* (6%). *E. faecium* and *E. hirae* have been frequently isolated from raw milk and dairy products (*25*-*27*). The presence of enterococci has for long been considered the result of poor hygiene during milking or handling (*2*), but these bacteria can be members of the normal microbiota of these products (*28*). Indeed, they play an important role in the ripening of several types of cheese *via* their lipolytic and proteolytic activities, and they contribute to flavour *via* the production of certain aromatic compounds (*29*).

### Technological characterisation of the isolates

#### Acidifying capacity of isolated strains

The ability of the strains to produce acid in commercial skimmed cow's milk was assessed by measuring the pH of the medium over 18 h (Table 2). All the isolates were slow acidifiers; none reduced the pH by more than 1 unit (*Lactobacillus* between 0.18 and 0.35, *Leuconostoc* 0.17 and 0.69, and *Enterococcus* isolates 0.24-0.78) in the first 6 h. After 18 h, the final pH varied from 6.14 by *Ln. mesenteroides* isolate LEY3 to 4.71 by *E. hirae* isolate LMA18. The *Lb. rhamnosus* isolates showed medium acidifying capacity and could be used as part of a mixed starter culture (*30*). Only four isolates (*Lb. rhamnosus* LEY15, *E. hirae* LMA16 and LMA18 and *E. faecium* LMA9) partially clotted the milk at the bottom of the tube after 18 h (Table 2). This low acidification activity of all the isolates indicates that they must be used in combination with strong acidifying starter cultures, for example of the species *Lc. lactis*, for the elaboration of dairy products.

**Table 2:** Acidification kinetics of the isolates from raw camel's milk in UHT skimmed cow's milk

	Incubation in UHT skimmed milk^a^		
Species/Strain		Time/h	
	6	18	18
	pH	pH	Milk clotting
*Ln. mesenteroides*			
LEY1	6.3±0.3	5.2±0.5	-
LEY2	6.1±0.1	5.3±0.3	-
LEY3	6.5±0.1	6.1±0.1	-
LEY4	6.37±0.08	6.0±0.1	-
LEY5	6.4±0.3	5.4±0.2	-
LEY9	6.50±0.06	5.6±0.2	-
LEY10	6.4±0.2	5.9±0.5	-
*Lb. rhamnosus*			
LEY14	6.5±0.2	5.3±0.3	-
LEY15	6.3±0.1	4.9±0.4	+/-
LEY16	6.6±0.1	5.6±0.5	-
LEY17	6.6±0.2	5.0±0.5	-
LEY18	6.6±0.2	4.8±0.2	-
LEY19	6.7±0.2	5.7±0.5	-
LEY20	6.5±0.1	5.3±0.6	-
*E. hirae*			
LMA16	6.0±0.2	4.98±0.09	+/-
LMA18	6.04±0.05	4.7±0.2	+/-
*E. faecium*			
LMA1	6.3±0.1	5.72±0.04	-
LMA2	6.44±0.08	5.6±0.5	-
LMA3	6.3±01	5.6±0.3	-
LMA4	6.3±0.3	5.6±0.3	-
LMA5	6.3±0.2	5.6±0.2	-
LMA6	6.1±0.2	5.2±0.5	-
LMA7	6.2±0.3	5.5±0.4	-
LMA8	6.49±0.09	5.8±0.2	-
LMA9	6.3±0.3	4.9±0.2	+/-
LMA10	6.6±0.2	6.01±0.03	-
LMA11	6.27±0.02	5.6±0.3	-
LMA12	6.3±0.2	5.4±0.2	-
LMA13	6.47±0.06	5.3±0.4	-
LMA14	6.3±0.2	5.0±0.6	-
LMA15	6.4±0.2	5.1±0.3	-
LMA17	6.4±0.2	5.8±0.2	-
LMA19	6.3±0.3	5.1±0.4	-
LMA20	6.3±0.2	5.1±0.3	-
LMA21	6.47±0.08	5.34±0.09	-

^a^pH of non-inoculated UHT skimmed milk was 6.77, +++ total clotting of milk, +/- partial clotting of milk at the bottom of the tube, UHT=ultra-high temperature

#### Proteolytic activity of isolated strains

**Fig. 1:**
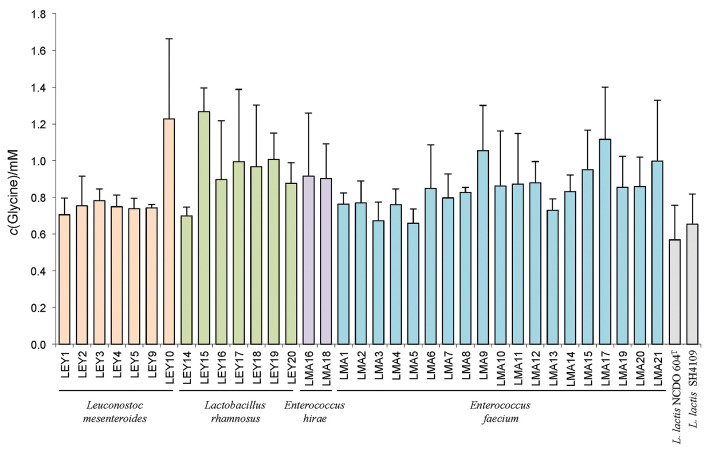
Proteolytic activity of strains isolated from Algerian raw camel's milk as determined by the OPA assay. Proteolytic activity was recorded as mmol of glycine released after incubation in skimmed cow's milk at 30 °C for 24 h, using a glycine calibration curve. *Lactococcus lactis* NCDO 604^T^ and *Lactococcus lactis* SH4109 strains were used as positive controls

Proteolytic activity against casein is a property of interest for any strain to be used as an adjunct culture (*31*). This ability of LAB strains to grow and develop in milk is essential (*32*). In the present work, proteolytic activity was examined qualitatively *via* growth on PCA supplemented with skimmed cow's milk, and quantitatively *via* the OPA assay. All the tested isolates showed a clarification halo on the supplemented PCA plates and were therefore considered proteolytic (data not shown). The OPA assay was more discriminating, revealing inter- and intra-species differences in proteolytic activity between the isolates (Fig. 1). All the lactobacilli had a proteolytic activity expressed in equivalents of glycinic acid (Gly) to about 1.00 mM), with isolate LEY14 showing the weakest activity (0.70±0.05) mM and isolate LEY15 the strongest (1.3±0.1) mM. *E. hirae* isolates LMA16 and LMA18 showed similar activity (0.9±0.3) and (0.9±0.2) mM, respectively. Among the *E. faecium* isolates, isolate LMA17 had the maximum activity, and the isolate LMA5 minimum (1.2±0.3) and (0.67±0.08) mM, respectively (denoting intraspecies variation). Intraspecies variation was also found among the *Leuconostoc* isolates. Isolate LEY10 showed notable activity with an equivalent of (1.2±0.4) mM; in contrast, none of the other *Leuconostoc* isolates exceeded 0.79 mM. As other authors report, milk proteolytic capacity appears to vary both within and among species isolated from natural sources (*33*, *34*). This ability is therefore strain-dependent (*28*, *35*, *36*). Interestingly, some isolates, such as *Lb. rhamnosus* LEY15 and *Ln. mesenteroides* LEY10, showed good proteolytic activity, suggesting they might have practical applications and be responsible for releasing peptides and amino acids responsible for the product texture and aroma (*37*, *38*). Some amino acids are involved in the production of aroma compounds, serving either directly or indirectly as precursors of aldehydes, acids, alcohols and esters (*35*), and thus contributing to the sensory profile of the end product (*5*).

#### Analysis of the citrate utilisation capability

The capacity of LAB to utilise the citrate present in milk is a desirable technological trait; its metabolism results in an excess of pyruvate that can be converted *via* α-acetolactate to diacetyl, acetoin and 2,3-butanediol - important flavour and aroma components of certain fermented dairy products (*39*). Table 3 shows that all the isolates of lactobacilli were positive for citrate utilisation, and in the case of *Leuconostoc*, only isolate LEY10 was unable to use it. The two *E. hirae* isolates were also able to utilise citrate, as were all but three *E. faecium* isolates (LMA1, LMA5 and LMA8) (Table 3).

**Table 3:** Citrate utilisation, and acetoin and dextran production by lactic acid bacteria isolated from Algerian camel's milk

Species/strain	Citrate utilisation	Acetoin production	Dextran production
*Ln. mesenteroides*			
LEY1	+	-	-
LEY2	+	-	+
LEY3	+	-	+
LEY4	+	-	+
LEY5	+	-	+
LEY9	+	-	+
LEY10	-	+	+
*Lb. rhamnosus*			
LEY14	+	+	-
LEY15	+	+	-
LEY16	+	+	-
LEY17	+	+	-
LEY18	+	+	-
LEY19	+	+	-
LEY20	+	+	-
*E. hirae*			
LMA16	+	+	-
LMA18	+	+	-
*E. faecium*			
LMA1	-	+	-
LMA2	+	+	-
LMA3	+	+	-
LMA4	+	+	-
LMA5	-	+	-
LMA6	+	+	-
LMA7	+	+	-
LMA8	-	+	-
LMA9	+	+	-
LMA10	+	+	-
LMA11	+	+	-
LMA12	+	+	-
LMA13	+	+	-
LMA14	+	+	-
LMA15	+	+	-
LMA17	+	+	-
LMA19	+	+	-
LMA20	+	+	-
LMA21	+	+	-

- negative producer, + positive producer

#### Acetoin production capability of isolated strains

Acetoin production, reflected by the presence of a pink ring in Clark and Lubs broth, was positive for all the tested enterococci, both the *E. hirae* and *E. faecium* isolates, all the *Lb. rhamnosus* isolates, and one *Leuconostoc* isolate (LEY10) (Table 3). Acetoin, which is produced *via* the catabolism of pyruvate, is responsible for the development of flavour and aroma. Enterococci and lactobacilli are the predominant genera able to produce it (*34*). Although *Ln. mesenteroides* LEY10, and *E. faecium* LMA1, LMA5 and LMA8 were unable to utilise citrate (Table 3), they seem to catabolise some of the pyruvate, produced from sugar, into acetoin. All the isolates of lactobacilli and enterococci produced acetoin in this work, and may play an important role in the development of the distinctive organoleptic properties of fermented camel dairy products.

#### Dextran production capability of isolated strains

Table 3 shows that only the *Leuconostoc* isolates produced dextran, with the exception of isolate LEY1. This would suggest the latter to be *Ln. mesenteroides* ssp. *cremoris*, a subspecies characterised by its non-production of this compound (*24*, *40*). The dextran-producing ability of the *Leuconostoc* isolates makes them candidates for use in secondary starters; dextran improves the appearance, stability and rheological properties of dairy products (*34*) and has potential health benefits (immunogenic properties, protection against gastric ulcers, improvement of digestive transit, and hypocholesterolaemic, antiviral and antitumoral activity, *etc.*) (*41*).

#### Analysis of the production of volatile compounds

Flavour development in dairy products is essentially an enzymatic process performed (mainly) by microorganisms (*35*). Lactose fermentation leads to the formation of pyruvate that can be further metabolised to ethanol, diacetyl, acetoin and acetaldehyde (*42*). By producing volatile compounds, such as ethanol (important in kefir and koumiss), diacetyl (important in butter, buttermilk and cheese) and acetaldehyde (important in yoghurt and buttermilk) (*5*), LAB contribute to the typical flavours of different dairy products.

The production of volatile compounds was assessed by HS/GC/MS (*17*). Eighteen major compounds were identified (Table 4). Wide variation was observable in the diversity and quantity of volatile compounds produced by the different isolates. The most common compounds produced were ethanol, acetaldehyde, methyl acetate, acetoin and acetic acid. Acetaldehyde was produced by all the isolates except *Ln. mesenteroides* LEY9 and LEY10. The production of acetoin in milk agreed with the results of the phenotypic test performed in Clark and Lubs medium. All the isolates of enterococci and lactobacilli gave positive results for both methods (HS/GC/MS, and Clark and Lubs medium) (Table 3 and Table 4). However, while *Ln. mesenteroides* LEY10 was positive in the phenotypic test, no acetoin was detected when it was grown in milk, indicating the need for confirmatory testing. The production of diacetyl was detected in all *Lb. rhamnosus* isolates and *E. hirae* LMA16, but not in *Ln. mesenteroides* nor *E. faecium* isolates. The LAB producing the least volatile compounds were the *Leuconostoc* isolates. This might be explained by the fact that they grow poorly in milk and that they must be combined with acid-producing lactococci in order to act as flavour producers in mixed starters (*12*, *35*, *43*). The ability of the *Lb. rhamnosus* isolates to produce flavour compounds such as diacetyl and acetoin suggests they could be used as adjunct cultures for developing cheese flavour (*44*).

**Table 4:** Volatile compound produced by the studied isolates from Algerian camel’s milk (HS/GC/MS analysis)

**Species/strain**	**Acetaldehyde**	**2-Propanone**	**2-Methyl propanal**	**Methyl acetate**	**2-Methyl butanal**	**3-Methyl butanal**	**2-Propanol**	**Ethanol**	**2,2,4,6,6-PMH**	**Diacetyl**	**Methyl butanoate**	**2-Methyl-1-propanol**	**Methyl hexanoate**	**2 or 3-Methyl1-butanol**	**Acetoin**	**Acetic acid**	**1,3 or 2,3-Butanol**	**Butanoic acid**
***Ln. mesenteroides***																		
**LEY1**	n.d.	331.0	n.d.	n.d.	n.d.	n.d.	342.7	3319.7	171.8	n.d.	n.d.	n.d.	n.d.	n.d.	n.d.	n.d.	n.d.	n.d.
**LEY2**	n.d.	n.d.	n.d.	n.d.	n.d.	n.d.	n.d.	6281.9	182.4	n.d.	n.d.	n.d.	n.d.	n.d.	n.d.	n.d.	n.d.	n.d.
**LEY3**	n.d.	n.d.	n.d.	n.d.	n.d.	n.d.	n.d.	13903.0	1874.9	n.d.	39.3	n.d.	33.1	n.d.	n.d.	n.d.	n.d.	n.d.
**LEY4**	n.d.	n.d.	n.d.	n.d.	n.d.	n.d.	n.d.	3296.0	1248.0	n.d.	41.3	n.d.	40.1	n.d.	n.d.	n.d.	n.d.	n.d.
**LEY5**	n.d.	n.d.	n.d.	n.d.	n.d.	n.d.	n.d.	6224.4	394.9	n.d.	25.5	n.d.	19.5	n.d.	n.d.	359.5	107.4	143.1
**LEY9**	72.6	n.d.	n.d.	n.d.	n.d.	n.d.	n.d.	3044.1	89.7	n.d.	3.7	n.d.	30.4	n.d.	n.d.	n.d.	n.d.	n.d.
**LEY10**	276.9	64.5	n.d.	n.d.	n.d.	n.d.	n.d.	5877.9	1818.9	n.d.	63.7	n.d.	51.8	n.d.	n.d.	111.0	n.d.	n.d.
***Lb. rhamnosus***																		
**LEY14**	15.0	n.d.	n.d.	50.7	n.d.	n.d.	n.d.	29.5	88.5	19.5	n.d.	n.d.	4.7	n.d.	122.5	86.4	n.d.	n.d.
**LEY15**	n.d.	n.d.	n.d.	36.8	n.d.	n.d.	n.d.	74.1	n.d.	13.5	n.d.	n.d.	n.d.	n.d.	130.5	128.6	n.d.	n.d.
**LEY16**	7.9	n.d.	n.d.	30.1	n.d.	n.d.	n.d.	28.1	81.4	21.5	n.d.	n.d.	n.d.	n.d.	128.9	161.1	n.d.	n.d.
**LEY17**	9.2	n.d.	n.d.	35.6	n.d.	n.d.	n.d.	27.8	63.8	8.1	n.d.	n.d.	4.7	n.d.	88.7	111.6	n.d.	n.d.
**LEY18**	8.8	n.d.	n.d.	37.7	n.d.	n.d.	n.d.	31.4	n.d.	18.7	n.d.	n.d.	n.d.	n.d.	96.3	94.1	n.d.	n.d.
**LEY19**	12.4	n.d.	n.d.	31.7	n.d.	n.d.	n.d.	56.6	49.1	24.5	n.d.	n.d.	n.d.	n.d.	132.8	144.9	n.d.	n.d.
**LEY20**	13.6	n.d.	n.d.	42.4	n.d.	n.d.	n.d.	30.0	89.0	20.4	n.d.	n.d.	6.6	n.d.	131.6	82.2	n.d.	n.d.
***E. hirae***																		
**LMA16**	7.6	n.d.	n.d.	73.7	n.d.	n.d.	n.d.	36.6	105.5	16.0	2.2	n.d.	n.d.	n.d.	33.6	3.8	n.d.	n.d.
**LMA18**	4.5	n.d.	n.d.	77.4	n.d.	n.d.	n.d.	36.4	95.3	n.d.	n.d.	n.d.	n.d.	n.d.	25.1	6.8	n.d.	n.d.
***E. faecium***																		
**LMA1**	32.3	n.d.	n.d.	45.7	n.d.	n.d.	n.d.	17.7	n.d.	n.d.	n.d.	n.d.	4.8	n.d.	51.2	86.5	n.d.	11.6
**LMA2**	51.7	n.d.	n.d.	n.d.	n.d.	n.d.	n.d.	70.2	95.0	n.d.	9.9	n.d.	21.4	n.d.	16.0	18.9	n.d.	n.d.
**LMA3**	27.2	n.d.	n.d.	18.4	n.d.	n.d.	n.d.	n.d.	n.d.	n.d.	n.d.	n.d.	3.4	n.d.	36.2	18.8	n.d.	n.d.-
**LMA4**	45.6	n.d.	n.d.	32.8	n.d.	n.d.	n.d.	n.d.	116.0	n.d.	8.1	n.d.	18.2	n.d.	56.6	29.7	n.d.	n.d.
**LMA5**	35.0	n.d.	n.d.	41.0	n.d.	n.d.	n.d.	n.d.	99.3	n.d.	5.8	n.d.	14.1	n.d.	53.1	32.7	n.d.	n.d.
**LMA6**	83.9	n.d.	n.d.	64.4	n.d.	n.d.	n.d.	275.4	118.2	n.d.	9.1	n.d.	20.3	n.d.	15.7	9.2	n.d.	n.d.
**LMA7**	85.5	n.d.	n.d.	15.7	n.d.	n.d.	n.d.	305.4	n.d.	n.d.	10.8	n.d.	21.2	n.d.	20.5	n.d.	n.d.	n.d.
**LMA8**	47.4	n.d.	n.d.	37.9	n.d.	n.d.	n.d.	13.6	n.d.	n.d.	8.5	n.d.	10.8	n.d.	34.5	n.d.	n.d.	n.d.
**LMA9**	147.4	n.d.	27.5	63.7	11.3	242.3	n.d.	30.5	n.d.	n.d.	12.7	8.8	24.6	126.1	94.0	68.9	n.d.	n.d.
**LMA10**	41.5	n.d.	n.d.	34.5	n.d.	n.d.	n.d.	n.d.	n.d.	n.d.	n.d.	n.d.	3.2	n.d.	34.0	73.5	n.d.	n.d.
**LMA11**	60.4	n.d.	n.d.	47.7	n.d.	n.d.	n.d.	18.0	130.7	n.d.	n.d.	n.d.	n.d.	n.d.	47.7	39.7	n.d.	n.d.
**LMA12**	35.1	n.d.	18.2	46.3	n.d.	n.d.	n.d.	8.6	104.7	n.d.	4.1	n.d.	14.8	n.d.	52.1	41.1	n.d.	n.d.
**LMA13**	73.8	n.d.	n.d.	66.3	n.d.	n.d.	n.d.	325.2	118.3	n.d.	8.1	n.d.	14.5	n.d.	6.7	53.1	n.d.	n.d.
**LMA14**	44.6	n.d.	n.d.	37.1	n.d.	n.d.	n.d.	101.2	65.5	n.d.	12.9	n.d.	21.3	n.d.	20.0	22.7	n.d.	n.d.
**LMA15**	44.6	n.d.	n.d.	24.1	n.d.	n.d.	n.d.	91.0	102.3	n.d.	15.0	n.d.	41.0	n.d.	20.5	n.d.	n.d.	n.d.
**LMA17**	56.8	n.d.	n.d.	21.6	n.d.	n.d.	n.d.	102.6	122.6	n.d.	12.7	n.d.	27.5	n.d.	17.2	5.8	n.d.	n.d.
**LMA19**	78.2	n.d.	n.d.	32.4	n.d.	n.d.	n.d.	338.4	121.7	n.d.	n.d.	n.d.	3.6	n.d.	9.2	14.2	n.d.	n.d.
**LMA20**	66.7	n.d.	n.d.	48.6	n.d.	n.d.	n.d.	318.9	136.5	n.d.	n.d.	n.d.	15.8	n.d.	6.7	n.d.	n.d.	n.d.
**LMA21**	40.9	n.d.	n.d.	21.6	n.d.	n.d.	n.d.	85.7	107.6	n.d.	15.3	n.d.	26.7	n.d.	17.6	n.d.	n.d.	n.d.

These values have no units, their area in the chromatogram was normalized as a percentage of the internal standard area, which was assigned to a value of 100%. PMH=pentamethyl heptane, n.d.=not detected

#### Antimicrobial substance production

The potential production of antimicrobial substances by the isolates was tested against a variety of indicator strains, including several LAB species, since bacteriocins produced by Gram-positive bacteria are most commonly active against closely related bacteria. Table 5 shows that none of the isolates inhibited the growth of *S. thermophilus* CNRZ 1066. The indicator *Lb. sakei* CECT 906^T^ was inhibited by all but one of the *Lb. rhamnosus* isolates, and by 42% of the *E. faecium* isolates, while *M. luteus* NCIMB 8166 was inhibited by 52% of the *Leuconostoc* isolates. *Lc. lactis* ssp. *cremoris* MG1363 was inhibited by 16% of the *E. faecium* isolates, *L. innocua* CECT 910^T^ was inhibited by 42% of the *E. faecium* isolates, and *S. thermophilus* LMD9 by 26% of the *E. faecium* isolates. Wider inhibition activity was observed for the *E. faecium* isolates, which inhibited the growth of four of the indicator strains (all except *S. thermophilus* CNRZ1066 and *M. luteus* NCIMB 8166). *E. faecium* LMA5 showed the strongest activity. LAB have a wide range of antimicrobial activities (*45*); *E. faecium* produces bacteriocin-like substances such as enterocins A and P, while *Ln. mesenteroides* produces leucocin (*46*). The present results agree with those reported in the literature, but further studies should test the capacity of these strains to inhibit the growth of pathogenic bacteria such as *Listeria monocytogenes.* They should also evaluate the potential of the isolates as adjunct cultures for improving food safety and shelf life.

**Table 5:** Antimicrobial activity expressed as the diameter of the inhibition halo

*d*(inhibition halo)/mm								
Species/Strain	*Lactobacillus sakei*C ECT 906^T^	*Listeria innocua* CECT 910^T^	*Lactococcus lactis* subsp. *cremoris* MG1363	*Micrococcus luteus* NCIMB 8166	*Streptococcus thermophilus LMD9*		*Streptococcus thermophilus CNRZ 1066*	
*Ln. mesenteroides*								
LEY1	n.d.	n.d.	n.d.	n.d.		n.d.		n.d.
LEY2	n.d.	n.d.	n.d.	n.d.		n.d.		n.d.
LEY3	n.d.	n.d.	n.d.	9.5		n.d.		n.d.
LEY4	n.d.	n.d.	n.d.	9.5		n.d.		n.d.
LEY5	n.d.	n.d.	n.d.	8.0		n.d.		n.d.
LEY9	n.d.	n.d.	n.d.	9.0		n.d.		n.d.
LEY10	n.d.	n.d.	n.d.	n.d.		n.d.		n.d.
*Lb. rhamnosus*								
LEY14	11.5	n.d.	n.d.	n.d.		n.d.		n.d.
LEY15	12.0	n.d.	n.d.	n.d.		n.d.		n.d.
LEY16	11.5	n.d.	n.d.	n.d.		n.d.		n.d.
LEY17	11.5	n.d.	n.d.	n.d.		n.d.		n.d.
LEY18	12.0	n.d.	n.d.	n.d.		n.d.		n.d.
LEY19	11.0	n.d.	n.d.	n.d.		n.d.		n.d.
LEY20	n.d.	n.d.	n.d.	n.d.		n.d.		n.d.
*E. hirae*								
LMA16	n.d.	n.d.	n.d.	n.d.		n.d.		n.d.
LMA18	n.d.	n.d.	n.d.	n.d.		n.d.		n.d.
*E. faecium*								
LMA1	24.0	16.5	12.0	n.d.		n.d.		n.d.
LMA2	n.d.	n.d.	n.d.	n.d.		n.d.		n.d.
LMA3	11.5	12.5	n.d.	n.d.		n.d.		n.d.
LMA4	11.5	11.5	n.d.	n.d.		10.0		n.d.
LMA5	23.0	15.5	10.0	n.d.		11.0		n.d.
LMA6	16.5	16.0	n.d.	n.d.		n.d.		n.d.
LMA7	15.0	15.5	n.d.	n.d.		12.0		n.d.
LMA8	n.d.	n.d.	n.d.	n.d.		n.d.		n.d.
LMA9	n.d.	n.d.	n.d.	n.d.		10.0		n.d.
LMA10	n.d.	n.d.	n.d.	n.d.		10.0		n.d.
LMA11	11.5	10.0	n.d.	n.d.		n.d.		n.d.
LMA12	22.0	16.0	10.0	n.d.		n.d.		n.d.
LMA13	n.d.	n.d.	n.d.	n.d.		n.d.		n.d.
LMA14	n.d.	n.d.	n.d.	n.d.		n.d.		n.d.
LMA15	n.d.	n.d.	n.d.	n.d.		n.d.		n.d.
LMA17	n.d.	n.d.	n.d.	n.d.		n.d.		n.d.
LMA19	n.d.	n.d.	n.d.	n.d.		n.d.		n.d.
LMA20	n.d.	n.d.	n.d.	n.d.		n.d.		n.d.
LMA21	n.d.	n.d.	n.d.	n.d.		n.d.		n.d.

n.d.=inhibitory activity not detected

#### Biogenic amine production capability

Dairy products accumulate the greatest diversity and quantity of biogenic amines (*47*). The consumption of food with elevated biogenic amine concentrations can lead to symptoms of intoxication. It should not, therefore, be allowed to accumulate to dangerous levels. One of the preventive measures that might be applied is the selection of strains for use in starter or co-starter cultures that are confirmed non-biogenic amine-producers (*6*). In the present work, the capacity to produce tyramine, histamine, putrescine and cadaverine was examined by UHPLC (*19*). Table 6 shows that all the *E. faecium* isolates produced tyramine. This is not surprising since tyramine production is a known species-dependent feature of *E. faecium* (*48*). The presence of biogenic amine-producing bacteria, such as enterococci, in the raw milk highlights the potential risk of allowing spontaneous fermentation, since as shown in this work, some harmful microorganisms could grow and accumulate toxic compounds (*49*). If *E. faecium* is to be used as an adjuvant culture, the balance between benefits and dangers must take into account the many factors that influence the accumulation of tyramine, such as the availability of tyrosine (due to casein proteolysis) and the presence of an acidic pH (*6*). Moreover, if a biogenic amine-producing strain also produces an antimicrobial substance, as is the case of *E. faecium* LMA5, it could dominate the microbiota and the risk of tyramine accumulation would be increased.

**Table 6:** Biogenic amines detected in different cultures of lactic acid bacteria isolated from Algerian raw camel’s milk

*c*(biogenic amine)/mM					
Species/strain	Tyramine	Histamine	Putrescine (AGDI)	Putrescine (ODC)	Cadaverine
*Ln. mesenteroides*					
LEY1	-	-	-	-	-
LEY2	-	-	-	-	-
LEY3	-	-	-	-	-
LEY4	-	-	-	-	-
LEY5	-	-	-	-	-
LEY9	-	-	-	-	-
LEY10	-	-	-	-	-
*Lb. rhamnosus*					
LEY14	-	-	-	-	-
LEY15	-	-	-	-	-
LEY16	-	-	-	-	-
LEY17	-	-	-	-	-
LEY18	-	-	-	-	-
LEY19	-	-	-	-	-
LEY20	-	-	-	-	-
*E. hirae*					
LMA16	-	-	-	-	-
LMA18	-	-	-	-	-
*E. faecium*					
LMA1	+	-	-	-	-
LMA2	+	-	-	-	-
LMA3	+	-	-	-	-
LMA4	+	-	-	-	-
LMA4	+	-	-	-	-
LMA6	+	-	-	-	-
LMA7	+	-	-	-	-
LMA8	+	-	-	-	-
LMA9	+	-	-	-	-
LMA10	+	-	-	-	-
LMA11	+	-	-	-	-
LMA12	+	-	-	-	-
LMA13	+	-	-	-	-
LMA14	+	-	-	-	-
LMA15	+	-	-	-	-
LMA17	+	-	-	-	-
LMA19	+	-	-	-	-
LMA20	+	-	-	-	-
LMA21	+	-	-	-	-

AGDI=agmatine deiminase route, ODC=ornithine decarboxylase route, + biogenic amine production,  - biogenic amine production not detected

### Cluster analysis of the isolates

**Fig. 2:**
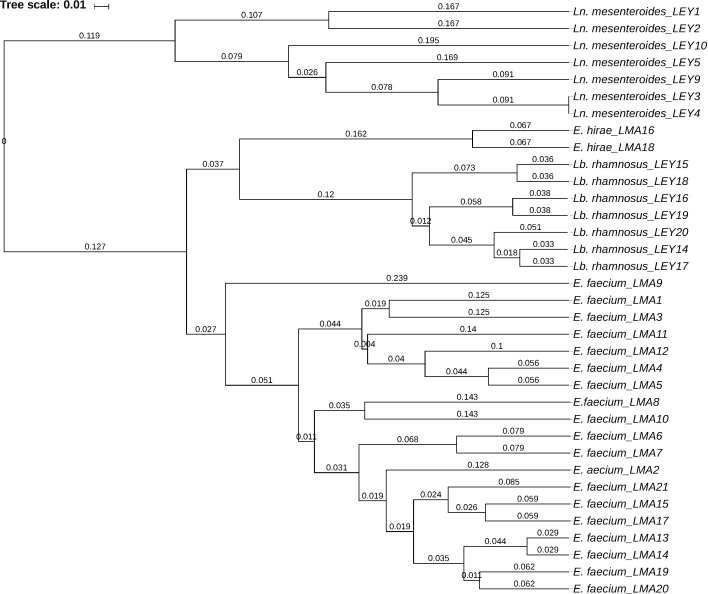
Dendrogram resulting from cluster analysis based on the 39 examined phenotypic characteristics of the 35 lactic acid bacteria isolates

 To typify the isolates and classify them as different strains, all 35 isolates were compared by clustering analysis (UPGMA) using the 39 examined phenotypic traits. Fig. 2 shows the resulting dendrogram. It should be noted that the analysis grouped together all the isolates of the same species. The results also indicate that all the isolates are different strains, except for *Ln. mesenteroides* LEY3 and LEY4, which clustered together. These may be understood as isolates of the same strain. The results of the cluster analysis based solely on phenotypic characteristics clearly showed the extent of intraspecies diversity. The wide phenotypic, biochemical and technological diversity among the isolated strains reflects the diversity of LAB in camel's milk (*2*). Indeed, it may be an excellent source of LAB with potential applications as adjunct cultures in the dairy industry for camel milk products, and perhaps beyond.

## CONCLUSIONS

Raw camel's milk produced in Algeria is a source of dairy LAB strains that might be used as adjunct cultures for the manufacture of camel, and perhaps other, dairy products. Of particular interest might be *Leuconostoc mesenteroides* LEY10, which showed good proteolytic activity and produced acetaldehyde and dextran in milk, all the *Lactobacillus rhamnosus* strains, which produced interesting flavour compounds such as diacetyl and acetoin and showed potential antimicrobial activities and *Enterococcus faecium* LMA5, which showed the strongest antimicrobial activity of all the isolates. The results of the cluster analysis based on the examined phenotypic characteristics clearly reveal the intraspecies diversity. This method might, therefore, be used for the typification of other isolates when no genetic information is available.
